# Seasonal Dynamics of Skin Microbiota and Metabolites in Transhumant-Grazed Altay Sheep

**DOI:** 10.3390/microorganisms14040901

**Published:** 2026-04-16

**Authors:** Xin Li, Zihang Qin, Haiyan Wang, Xinyu Tao, Jiangtao Xia, Yukang Zhao, Pengfei Yi, Yunxiao Ma, Xinhao Wang, Xuelian Ma, Na Li, Qi Zhong, Gang Yao

**Affiliations:** 1 College of Veterinary Medicine, Xinjiang Agricultural University, Urumqi 830052, China; 320220061@xjau.edu.cn (X.L.); 320232860@stu.xjau.edu.cn (Z.Q.); 18841658067@163.com (X.T.); 18997798685@163.com (J.X.); 18809066861@163.com (Y.Z.); y448398022@163.com (P.Y.); 18999838116@sina.cn (Y.M.); m13889823869@163.com (X.W.); maxuelian@xjau.edu.cn (X.M.); nali@edu.xjau.cn (N.L.); 2 Center of Diagnosis and Control for Animal Diseases in Aletai Prefecture, Aletai 836500, China; wanghaiy1977@163.com; 3 Institute of Veterinary Research, Xinjiang Academy of Animal Sciences, Urumqi 830011, China; 13932533005@163.com

**Keywords:** skin microecology, cutaneous barrier, functional pathways

## Abstract

To explore the seasonal variation patterns of the skin microecology of Altay sheep under transhumant grazing conditions, skin swabs were collected from 60 free-grazing Altay sheep at seasonal transition nodes in the Altay region. Metagenomic sequencing combined with untargeted metabolomics was used to characterize their bacterial community structure, functional pathways, and metabolite profiles. The results showed that the skin microecology of Altay sheep presented obvious seasonal variation patterns. In spring, 35 of the 39 highly abundant bacteria were environmentally derived, five proliferation-related pathways were significantly enriched, and the levels of five metabolites associated with microbial community regulation and skin barrier defense were elevated. In summer, the abundance of three skin symbiotic bacteria increased, the activities of eight pathways mainly related to biofilm formation were significantly enhanced, and the contents of five metabolites primarily associated with membrane lipid homeostasis and selective bacteriostasis increased. In autumn, the abundances of nine radiation-resistant and cold-tolerant strains increased, together with the elevated abundance of two opportunistic pathogens; five repair-related pathways were active, and the levels of four anti-inflammatory and repair-associated metabolites were synchronously increased. In winter, the abundance of two cold-tolerant strains increased, the activities of pathways related to nitrogen metabolism and energy synthesis were enhanced, and one lignan compound was identified as the key metabolite. These findings elucidate the seasonal dynamic patterns of the skin microecology of Altay sheep and provide a theoretical basis for research on the adaptive mechanisms and seasonal health management of Altay sheep and other sheep in alpine regions.

## 1. Introduction

The Altay region is located at the northernmost tip of the Xinjiang Uygur Autonomous Region, at the junction of the southern foot of the Altai Mountains and the northern margin of the Junggar Basin. This region is dominated by desert steppe and has approximately 9.33 million hectares (140 million mu) of natural pastures, serving as a major traditional livestock production area in China. This region has a temperate continental arid climate characterized by marked seasonal climatic variations. The sheep industry is among the local pillar industries, whose feeding system is dominated by four-season transhumant nomadic grazing [1]. From winter to spring, the sheep graze in the Irtysh River Valley, which is characterized by plain valleys; in summer, they graze in the Gobi pastures with desert Gobi landforms; and in autumn, they graze in the mountain forest pastures with abundant forage. The predominant breed raised is the Altay sheep, an excellent indigenous sheep breed in China with outstanding traits of large body size, early maturity, superior rough forage tolerance, and strong disease resistance. With fine and tender meat and a unique flavor, Altay sheep have gradually become a representative breed in the high-end mutton market, with a continuously expanding breeding scale [2,3].

The skin is the largest organ of the mammalian body in terms of surface area. It not only forms the first physical barrier against the external environment but also harbors a complex skin microecosystem [4,5]. Commensal microbial communities colonizing the skin surface play critical roles in maintaining skin barrier function, resisting invasion by pathogenic microorganisms, and regulating host immune responses [6]. Moreover, metabolites present on the skin surface are key molecular components for maintaining skin homeostasis, as they participate in multiple physiological processes, including barrier lipid synthesis, pH regulation, antimicrobial peptide secretion, and immune signal transduction [7,8]. The synergistic interaction between the skin microbiota and metabolites constitutes a vital microecological defense system for the host [9].

At present, most research on livestock and poultry microecology has focused on the intestinal tract, while reports on the seasonal dynamics of sheep skin microecology under a four-season transhumant grazing system are lacking. This grazing regime periodically exposes Altay sheep to environments with dramatic fluctuations in temperature, humidity, vegetation composition, and ultraviolet radiation, and these varying environmental factors have been confirmed to shape the structural characteristics of host skin microecology [10,11]. To address this research gap and elucidate the seasonal variation patterns of Altay sheep skin microecology, we conducted this study on traditionally transhumant-grazed Altay sheep in the Altay region of Xinjiang. Using an integrated approach of metagenomic sequencing and untargeted metabolomics [8], we characterized the seasonal variation features of the dominant bacterial species composition, functional metabolic pathways, and skin metabolite profiles of Altay sheep across four seasons. This study provides a theoretical basis for exploring the seasonal adaptation mechanisms between the ovine skin microbiome and the host, as well as a scientific reference for precision feeding management and disease prevention and control of Altay sheep.

This study aimed to investigate the seasonal patterns and differences in the skin bacterial community structure, metabolic pathways, and metabolite composition of Altay sheep under natural grazing conditions across four seasons. It provides a theoretical basis for research on the skin microecological adaptation mechanisms and seasonal health management of sheep in the Altay region, as well as alpine, arid, and semi-arid pastoral areas worldwide.

We hypothesized that the dramatic seasonal environmental changes under transhumant grazing would significantly reshape the skin microbial community structure, functional metabolic pathways, and metabolite composition of Altay sheep and that such seasonal dynamics serve as a crucial adaptive mechanism for sheep to adapt to alpine and arid pastoral environments and maintain skin microecological homeostasis as well as host health.

## 2. Materials and Methods

### 2.1. Materials

#### 2.1.1. Experimental Animals and Sample Collection

Experimental Animals: A total of 60 free-grazing Altay sheep with equal numbers of males and females were selected as experimental subjects. All rams were castrated. Ewes were bred around November, underwent a 5-month gestation period spanning spring and winter, and lambed around April; sample collection in this study was carried out at an interval of at least 15 days away from both breeding and gestation. The sheep had a body weight of 36.8 ± 3.8 kg and an age of 36 ± 1 months. All animals were in good clinical health and had not received any pharmacological treatment within the preceding 8 months prior to sample collection.

Exclusion criteria: Animals with visible skin diseases, wounds, or parasitic infestations, those that had received any medication within 8 months prior to sampling, and those showing signs of systemic illness or stress.

Seasonal Grazing Conditions of Pastoral Areas: The experimental sheep underwent year-round four-season transhumant rotational grazing, with grazing locations and geomorphological characteristics for each season as follows: In winter (December to February of the following year) and spring (March to May), the flock grazed in the Irtysh River Valley, dominated by plain valley landforms; in summer (June to August), they were transferred to Gobi pastures characterized by Gobi Desert landforms; and in autumn (September to November), grazing was conducted in alpine pastures with mountainous landforms. In this study, the 60 experimental sheep were assigned to the spring group at the spring sampling time point, the summer group at the summer sampling time point, the autumn group at the autumn sampling time point, and the winter group at the winter sampling time point. No additional control groups were set up in this experiment. The detailed sampling times, grazing geomorphology, and corresponding climatic conditions for each season are shown in Table 1. Meteorological information in this study was obtained from the China Meteorological Data Service Centre (http://data.cma.cn/). accessed on 25 April 2023; 10 June 2023; 15 September 2023; 15 December 2023.

#### 2.1.2. Main Instruments and Equipment

For the metagenomic sequencing experiments, a microcentrifuge (Fresco 17, Thermo Fisher Scientific Inc., Waltham, MA, USA), thermomixer (ThermoMixer C, Thermo Fisher Scientific Inc., Waltham, MA, USA), ultrasonic fragmenter (LE220R-plus, Covaris Inc., Woburn, MA, USA), automated nucleic acid library preparation system (Biomek i7 Hybrid, Beckman Coulter, Inc., Brea, CA, USA), real-time fluorescent quantitative PCR system (CFX96, Bio-Rad Laboratories, Inc., Hercules, CA, USA), automated capillary electrophoresis system (Fragment Analyzer 5400, Agilent Technologies, Inc., Santa Clara, CA, USA), and high-throughput sequencing platform (NovaSeq 6000, Illumina Inc., San Diego, CA, USA) were used.

For untargeted metabolomics experiments, a mass spectrometer (Q Exactive HF/Q Exactive HF-X; Thermo Fisher Scientific, Inc., Waltham, MA, USA), an ultrahigh-performance liquid chromatograph (Vanquish UHPLC; Thermo Fisher Scientific, Inc., Waltham, MA, USA), a chromatographic column (Hypersil Gold C18; Thermo Fisher Scientific, Inc., Waltham, MA, USA), and a low-temperature centrifuge (D3024R; Scilogex LLC, Rocky Hill, CT, USA) were used.

#### 2.1.3. Main Reagents

For metagenomic sequencing, the following reagents were used: cetyltrimethylammonium bromide (CTAB) DNA extraction reagent (C5670, Sigma-Aldrich Trading Co., Shanghai, China); PCR buffer (M0531S, New England Biolabs, Beijing, China); DNA polymerase (M0530S, New England Biolabs, Beijing, China); and a library preparation kit (E7645S, Illumina Inc., San Diego, CA, USA).

For untargeted metabolomics, methanol (A456-4, Thermo Fisher Scientific Inc., Waltham, MA, USA), water (1.15333.2500, Thermo Fisher Scientific Inc., Waltham, MA, USA), formic acid (A117-50, Thermo Fisher Scientific Inc., Waltham, MA, USA), and ammonium acetate (A114-50, Thermo Fisher Scientific Inc., Waltham, MA, USA) were used.

### 2.2. Methods

#### 2.2.1. Sample Collection

At each seasonal sampling time point, samples were collected from these 60 experimental sheep, with three skin swabs obtained per animal (one for detection and two reserved as sample backups). Sample collection was performed in accordance with the method described by Manus et al. [12]. Prior to sampling, the sampling area was gently wiped with a sterile normal saline cotton ball to remove surface dust and large particulate contaminants, and swab collection was conducted only after the sampling area was completely air-dried. Sterile cotton swabs were used to wipe four anatomical sites for sampling, including the neck, chest, inguinal region, and gluteal region, with a sampling area of approximately 3 cm × 3 cm per site. Each site was wiped 30 times to ensure sufficient sample biomass. After collection, the swab head was immediately clipped into a cryopreservation tube, snap-frozen in liquid nitrogen, and then transferred to a −80 °C ultralow-temperature freezer for storage until sample processing completed within 3 days of collection. All the samples were uniformly numbered and labeled in accordance with the rules of animal ID, sampling site, and sampling season. All DNA extraction and library preparation procedures were performed in a dedicated clean laboratory with separate pre-PCR and post-PCR areas. All work surfaces were decontaminated with 70% ethanol and UV-irradiated before use. Filtered pipette tips and DNase/RNase-free tubes were used throughout. Negative controls (sterile swabs processed through the entire workflow) were included in each batch. No detectable bacterial DNA was observed in any negative control samples, confirming the absence of contamination [12].

#### 2.2.2. Experimental Design

In this study, metagenomic sequencing was used to analyze the skin bacterial community, including alpha diversity (Chao1, observed-features, Shannon, Simpson, and Faith’s PD indices), beta diversity (principal coordinate analysis, PCoA, based on the Bray–Curtis distance), and species composition and relative abundance analysis at the phylum, genus, and species taxonomic levels. Moreover, untargeted metabolomics was performed for the systematic detection of skin metabolite profiles. By combining partial least squares discriminant analysis (PLS-DA) and random forest models, we identified bacterial species, Kyoto Encyclopedia of Genes and Genomes (KEGG) functional metabolic pathways, and characteristic metabolites whose abundance significantly differed across seasons.

#### 2.2.3. Metagenomic Sequencing

Genomic DNA was extracted from skin swab samples using the CTAB method. Briefly, 1000 μL of CTAB lysis buffer (containing lysozyme at a final concentration of 100 μg/mL) was added to a 2.0 mL centrifuge tube, followed by the sample (200 μL of swab eluate). After thorough mixing, the mixture was incubated in a 65 °C water bath for 60 min, with inversion and mixing performed every 15 min to ensure complete sample lysis. After centrifugation was performed at 12,000× *g* for 10 min at 4 °C, the supernatant was transferred to a new tube, and an equal volume of phenol (pH 8.0):chloroform:isoamyl alcohol (25:24:1) mixture was added. After thorough mixing by inversion, centrifugation was repeated at 12,000× *g* for 10 min at 4 °C. The supernatant was aspirated into a 1.5 mL centrifuge tube, 0.7 volumes of isopropanol were added, and the mixture was gently mixed and placed at −20 °C for precipitation for at least 30 min. After another centrifugation at 12,000× *g* for 10 min at 4 °C, the supernatant was discarded, and the pellet was washed twice with 1 mL of 75% ethanol and air-dried at room temperature. An appropriate volume of nuclease-free water was added to reconstitute the DNA, followed by incubation at 55–60 °C for 10 min to facilitate dissolution. One microliter of RNase A (10 mg/mL) was added, and the mixture was incubated at 37 °C for 15 min to remove RNA contamination.

DNA concentration, integrity, and purity were assessed using an Agilent 5400 Fragment Analyzer (OD260/280 ratio of 1.8–2.0; OD260/230 ratio ≥ 1.8). All the samples passed quality control before being entered into the library construction workflow. Genomic DNA was randomly sheared into ~350 bp fragments using a Covaris ultrasonic disruptor. Subsequently, end repair, 3′ A-tailing, ligation of Illumina sequencing adapters, bead-based fragment size selection, and PCR amplification were performed sequentially according to the manufacturer’s protocol for the NEBNext Ultra DNA Library Prep Kit (New England Biolabs, Ipswich, MA, USA) for Illumina. After the PCR amplicons were purified with AMPure XP beads, the fragment size distribution and integrity of the libraries were re-evaluated using an Agilent 5400 Fragment Analyzer, and the effective concentration of the libraries was accurately quantified by real-time quantitative PCR (qPCR) (diluted to 1.5 nM). Libraries were pooled equimolarly according to their effective concentration and target sequencing data volume, and high-throughput sequencing was performed on the Illumina NovaSeq 6000 platform using a paired-end 150 bp (PE150) sequencing strategy. The average number of reads per sample was 5.8 ± 0.6 million, and the percentage of host DNA removal was approximately 92.3 ± 3.1%. The sequencing quality criteria were set as follows: Q20 ≥ 97% and Q30 ≥ 92%, ensuring high-quality sequencing data for subsequent analysis.

For the bioinformatics analysis of metagenomic data, Kraken2 (v2.1.2) was used for taxonomic classification, and Bracken (v2.6.0) was employed to correct the taxonomic abundance. The NCBI RefSeq bacterial genome database (release 214) was used as the reference database for taxonomic annotation. For the statistical analysis of differential taxa and functional pathways, the Benjamini–Hochberg method was applied for correction of multiple comparisons to control the false discovery rate (FDR) at 0.05, ensuring the reliability of the statistical results.

It is important to note that this study employed a culture-independent metagenomic approach to characterize the skin bacterial community. Therefore, pure bacterial isolates were not obtained, and conventional antibiotic susceptibility testing could not be performed. The bacterial species were identified through DNA sequencing and bioinformatic analysis, providing information about community composition and functional potential rather than the phenotypic characteristics of individual isolates.

#### 2.2.4. Untargeted Metabolomics Analysis

Metabolite Extraction: Skin swabs were placed in 1.5 mL centrifuge tubes, 1000 μL of prechilled 80% (*v*/*v*) methanol aqueous solution was added, and the mixture was vortexed for 30 s and incubated on ice for 5 min, followed by centrifugation at 15,000× *g* for 20 min at 4 °C. The supernatant was transferred to a new centrifuge tube, lyophilized to dry powder using a vacuum freeze dryer, and reconstituted with 100 μL of 10% (*v*/*v*) methanol aqueous solution. After another centrifugation at 15,000× *g* for 5 min at 4 °C, the supernatant was collected for LC–MS analysis. Quality control (QC) samples, prepared by pooling equal volumes of all samples, were processed in parallel with each batch of experiments to monitor instrument operational stability and data quality. A coefficient of variation (CV) of less than 15% was used to ensure the stability of the detection system.

Chromatographic Conditions: Chromatographic separation was performed using a Hypersil Gold C18 reversed-phase column, with a column temperature of 40 °C, an injection volume of 5 μL, and a flow rate of 0.2 mL/min. Mobile phase A was 0.1% (*v*/*v*) formic acid in water, and mobile phase B was methanol. The gradient elution program was as follows: initial isocratic elution with 98% A and 2% B from 0 to 1.5 min; a linear gradient adjusted to 15% A and 85% B at 3 min; a continuous linear gradient to 100% B at 10 min; and a rapid return to the initial ratio (98% A, 2% B) at 10.1 min, which was maintained until 12 min for column equilibration, with a total run time of 12 min.

Mass Spectrometric Conditions: Mass spectrometric detection was performed using an electrospray ionization (ESI) source, with independent sample injection and acquisition in positive and negative ion modes, respectively. The mass spectrometry parameters were set as follows: scan range, *m*/*z* 100–1500; spray voltage, 3.5 kV; sheath gas flow rate, 35 arb; auxiliary gas flow rate, 10 arb; ion transfer tube temperature, 320 °C; S-lens RF level, 60; and auxiliary gas heater temperature, 350 °C. Tandem mass spectrometry (MS/MS) was performed in data-dependent acquisition (DDA) mode. The raw data were collected using Xcalibur (v 4.7) software.

Data Preprocessing and Metabolite Identification: Raw data files were converted to mzXML format using ProteoWizard (v 3.0) software and then imported into the XCMS (v 4.1.3) software package for peak detection, peak extraction, and peak alignment. Before filtering, a total of 4236 features were detected, and after filtering to remove noise, interference peaks, and background ions, 3872 valid features were retained. The feature peak matrix of each sample was integrated using the retention time (RT) and mass-to-charge ratio (*m*/*z*) as matching parameters. Solvent blank samples were used to filter background ions and remove abiotic interference peaks. Total ion current (TIC) normalization was subsequently performed on the raw peak areas to obtain the relative peak area of each metabolic feature. For metabolite identification, the acceptance criteria were set as follows: the mass deviation does not exceed 10 ppm, and the MS/MS fragment matching degree is not less than 80%. The confidence level of metabolite annotation was in accordance with the Metabolomics Society standards [13], including level 1 (confirmed by authentic standards) and level 2 (putative identification). For metabolite identification, a mass deviation threshold of 10 ppm was set, and MS/MS fragment information was aligned with the Human Metabolome Database (HMDB) and METLIN database, combined with adduct ion type information. The identification results were reported in accordance with the grading standards recommended by the Metabolomics Society. The identified metabolites were annotated against the KEGG, HMDB, and LIPID MAPS databases to obtain metabolite classification information and biochemical pathway assignments.

#### 2.2.5. Data Processing and Statistical Analysis

Metagenomic Sequencing Data Analysis: Quality control preprocessing of raw metagenomic sequencing data was performed using KneadData (v 0.12.4) software: adapter sequences and low-quality bases (quality score < 20) were trimmed using Trimmomatic (v 0.40) [14], and sequences with a final length less than 50 bp were filtered out. The filtered sequences were aligned to the sheep reference genome (*Ovis aries*, *Oar_v3.1*, NCBI RefSeq: GCF_000298735.2) using Bowtie2 (v 2.5.5) [15] to remove host-derived sequences, and non-host sequences were retained for subsequent analysis. Quality control performance was evaluated using FastQC (v 0.12.1). Kraken2 was used to align the quality-controlled valid sequences against bacterial sequences in the NCBI NT nucleotide database and RefSeq whole-genome database, followed by Bracken [16] for quantitative estimation of the actual relative abundance of bacterial species in each sample based on the Bayesian re-estimation method.

For alpha diversity analysis, Chao1, observed-features, Shannon, Simpson, and Faith’s PD indices were calculated on the basis of the species abundance matrix. The Kruskal–Wallis test was used to analyze differences in alpha diversity among different seasonal groups, as the data did not conform to a normal distribution. For beta diversity analysis, a Bray–Curtis dissimilarity matrix was used, and the differences in community structure between seasons were visualized by PCoA, with the significance of intergroup differences tested by permutational multivariate analysis of variance (PERMANOVA) [17]. The PERMANOVA analysis included F-value, R^2^ value (reflecting variance proportion from grouping), and adjusted *p*-values (controlling false discovery rate), ensuring reliable intergroup difference analysis. The functional annotation of quality-controlled and host-depleted sequences was performed using HUMAnN2 (v 2.8.2) [18] software, with DIAMOND (v 2.1.10) [19] as the alignment engine to align sequences against the UniRef90 protein database. KEGG pathway functional annotation information and a relative abundance matrix were constructed according to the correspondence between UniRef90 IDs and each functional database.

Untargeted Metabolomics Data Analysis: PLS-DA was performed on the metabolomics data using the ropls package in R software (v 4.4.3) [20] to visualize the season-specific clustering characteristics of skin metabolite profiles across the four seasons, and model stability was evaluated by 7-fold cross-validation (R2Y and Q2 parameters) [21]. The random forest algorithm was used for further screening of differentially abundant metabolites in the untargeted metabolomics data [22] to identify key metabolites with significant discriminatory contributions across seasons [23]. The analysis was implemented using the randomForest package (v 4.7) in R software: A multiclass random forest model was constructed with seasonal grouping as the response variable and the relative peak area of each metabolic feature as the input features. The parameter settings were as follows: number of decision trees (ntree) = 500, number of features randomly sampled at each node (mtry) = the square root of the total number of features, and model classification performance was evaluated by 10-fold cross-validation. The contribution of each metabolite was ranked by the mean decrease accuracy (MDA) value, and key differentially abundant metabolites were screened in combination with the significance of interseason differences (*p* < 0.05).

Differential Data Analysis: All the data are expressed as the mean ± standard deviation (mean ± SD). One-way analysis of variance (one-way ANOVA) was used to test intergroup differences in the relative abundance of skin bacterial species (phyla with mean relative abundance > 0.1% and genera and species with mean relative abundance > 0.5% in each group) and skin microbial functional prediction results (top 0.1% of level 3 KEGG pathways by mean relative abundance), with Tukey’s HSD test for post hoc multiple comparisons [24,25]. All the statistical analyses and visualizations were performed using GraphPad Prism 10.0.4. A *p* value < 0.05 was regarded as indicating statistical significance, and *p* < 0.001 was regarded as indicating extreme statistical significance. Statistical significance was defined as follows: * *p* < 0.05, ** *p* < 0.01 and *** *p* < 0.001, and these significance indicators were directly added to the corresponding figures to intuitively reflect intergroup differences.

## 3. Results

### 3.1. Metagenomic Sequencing

**Figure 1:**
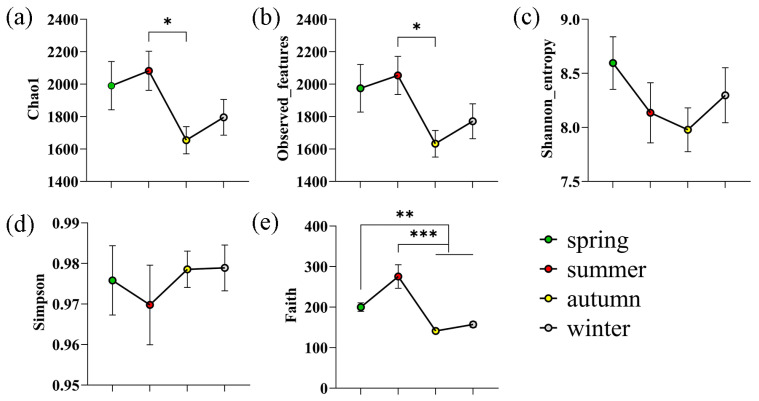
Alpha diversity indices. The results are presented as “mean ± standard deviation”, * indicates *p* < 0.05 (significant difference), ** indicates *p* < 0.01 (highly significant difference), *** indicates *p* < 0.001. (**a**) Chao1; (**b**) Observed-features; (**c**) Shannon_entropy; (**d**) Simpson; (**e**) Faith.

Seasonal changes in the alpha diversity of the sheep skin microbiota across the four seasons were assessed. As shown in Figure 1a,b, the Chao1 and observed-features indices of the skin microbiota of the sheep in summer were significantly greater than those in autumn (*p* < 0.05). As shown in Figure 1e, the Faith’s PD indices of skin microbiota in spring and summer were extremely significantly higher than those in autumn and winter (*p* < 0.01 and *p* < 0.001, respectively). As shown in Figure 1c,d, Shannon entropy and Simpson index showed no significant differences.

**Figure 2:**
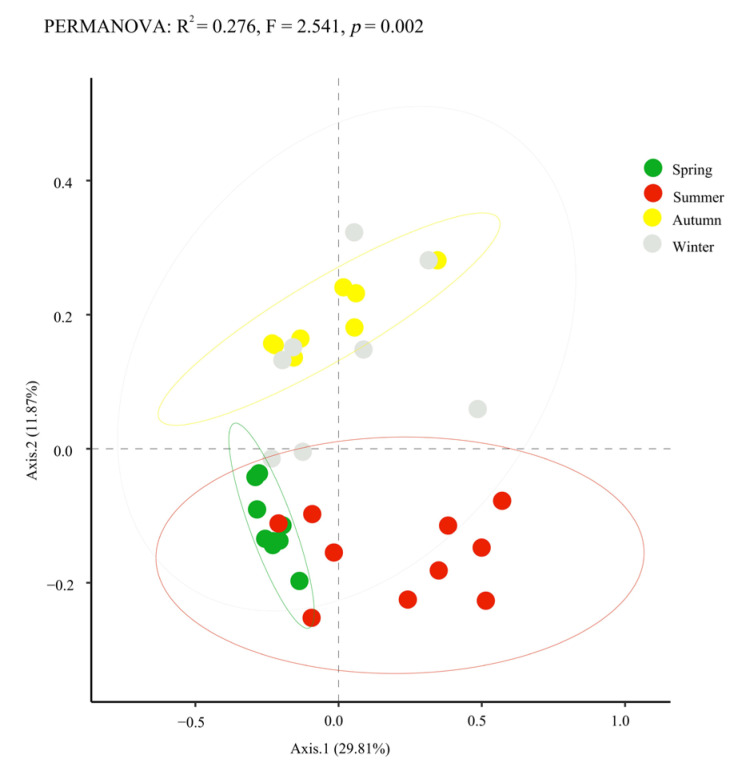
PCoA based on the Bray–Curtis distance.

Principal coordinate analysis (PCoA) was performed based on the Bray–Curtis distance to evaluate the beta diversity of the bacterial community structure on sheep skin. As shown in Figure 2, the distribution of confidence ellipses across the four seasons revealed that the spring group was concentrated in the lower-left quadrant, the summer group in the upper-right region, and the autumn and winter groups scattered in the upper-middle area. Furthermore, permutational multivariate analysis of variance (PERMANOVA) for similarity analysis demonstrated that significant differences existed among all groups, as presented in Table 2 (*p* < 0.05).

**Figure 3:**
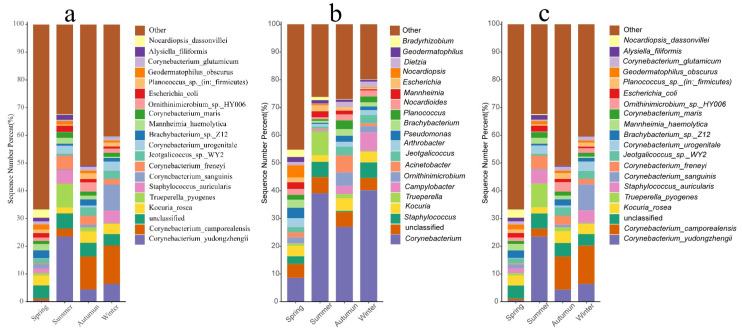
Composition of the top 20 phyla, genera, and species in terms of relative abundance. Note: (**a**): Bar chart of the top 20 phyla by relative abundance. (**b**): Bar chart of the top 20 genera by relative abundance. (**c**): Bar chart of the top 20 species by relative abundance.

Phylum-level composition: A total of 42 bacterial phyla were detected in the sheep skin microbiota across the four seasonal groups. The 20 most abundant phyla, as shown in Figure 3a, were *Firmicutes*, *Actinobacteriota*, *Bacteroidota*, *Proteobacteria*, *Spirochaetota*, *Campilobacterota*, *Fusobacteriota*, *Chloroflexi*, *Patescibacteria*, *Acidobacteriota*, *Gemmatimonadota*, *Verrucomicrobia*, *Desulfobacterota*, *Cyanobacteria*, *Myxococcota*, *Deferribacterota*, *Fibrobacterota*, *Planctomycetota*, *Deinococcota*, and *Bdellovibrionota*.

Genus-level composition: A total of 1516 bacterial genera were detected in the skin microbiota of the sheep across the four seasonal groups. The 20 most abundant genera, as shown in Figure 3b, were *Corynebacterium*, unclassified, *Staphylococcus*, *Kocuria*, *Trueperella*, *Campylobacter*, *Ornithinimicrobium*, *Acinetobacter*, *Jeotgalicoccus*, *Arthrobacter*, *Pseudomonas*, *Brachybacterium*, *Planococcus*, *Nocardioides*, *Mannheimia*, *Escherichia*, *Nocardiopsis*, *Dietzia*, *Geodermatophilus*, *Bradyrhizobium*, and others.

**Figure 4:**
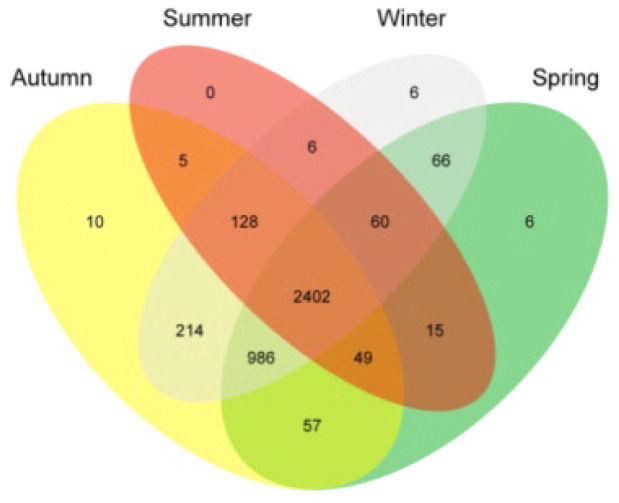
Species statistics by season.

Species-level composition: A total of 4010 bacterial species were detected in the skin microbiota across the four seasons (Figure 4). Specifically, 6 unique bacterial species were observed exclusively in spring, no unique species were found in summer, 10 unique species were present only in autumn, and 6 unique species were detected solely in winter. The 20 most abundant bacteria shown in Figure 3c were *Corynebacterium yudongzhengii*, *Corynebacterium camporealensis*, unclassified, *Kocuria rosea*, *Trueperella pyogenes*, *Staphylococcus auricularis*, *Corynebacterium sanguinis*, *Corynebacterium freneyi*, *Jeotgalicoccus* sp. WY2, *Corynebacterium urogenitale*, *Brachybacterium* sp. Z12, *Mannheimia haemolytica*, *Corynebacterium maris*, *Ornithinimicrobium* sp. HY006, *Escherichia coli*, *Planococcus* sp. (*Firmicutes*), *Geodermatophilus obscurus*, *Corynebacterium glutamicum*, *Alysiella filiformis*, and *Nocardiopsis dassonvillei*.

**Figure 5:**
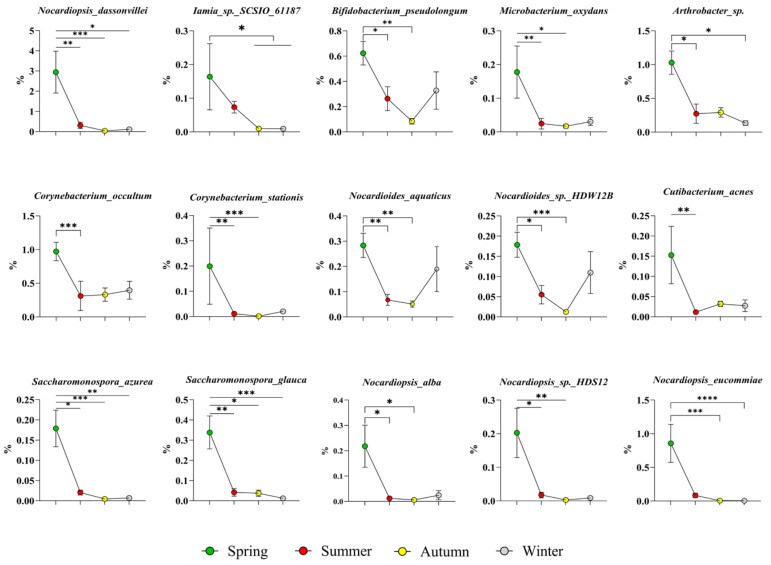
High-abundance bacteria of the phylum Actinobacteriota in spring. The results are presented as “mean ± standard deviation”, * indicates *p* < 0.05 (significant difference), ** indicates *p* < 0.01 (highly significant difference), *** indicates *p* < 0.001, **** indicates *p* < 0.0001.

High-abundance bacteria of the phylum *Actinobacteriota* in spring: As shown in Figure 5, 15 species were identified, namely, *Nocardiopsis dassonvillei*, *Iamia* sp. SCSIO_61187, *Bifidobacterium pseudolongum*, *Microbacterium oxydans*, *Arthrobacter* sp., *Corynebacterium occultum*, *Corynebacterium stationis*, *Nocardioides aquaticus*, *Nocardioides* sp. HDW12B, *Cutibacterium acnes*, *Saccharomonospora azurea*, *Saccharomonospora glauca*, *Nocardiopsis alba*, *Nocardiopsis* sp. HDS12, and *Nocardiopsis eucommiae*.

**Figure 6:**
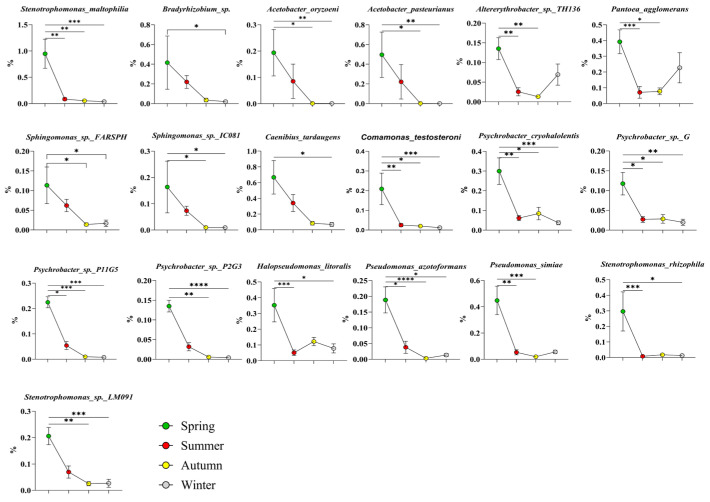
High-abundance bacteria of the Protobacteria in spring. The results are presented as “mean ± standard deviation”, * indicates *p* < 0.05 (significant difference), ** indicates *p* < 0.01 (highly significant difference), *** indicates *p* < 0.001, **** indicates *p* < 0.0001.

High-abundance bacteria of the phylum *Proteobacteria* in spring: As shown in Figure 6, 19 species were identified, namely, *Stenotrophomonas maltophilia*, *Bradyrhizobium* sp., *Acetobacter oryzoeni*, *Acetobacter pasteurianus*, *Altererythrobacter* sp. TH136, *Pantoea agglomerans*, *Sphingomonas* sp. FARSPH, *Sphingomonas* sp. IC081, *Caenibius tardaugens*, *Comamonas testosteroni*, *Stenotrophomonas* sp. LM091, *Psychrobacter cryohalolentis*, *Psychrobacter* sp. G, *Psychrobacter* sp. P11G5, *Psychrobacter* sp. P2G3, *Halopseudomonas litoralis*, *Pseudomonas azotoformans*, *Pseudomonas simiae*, and *Stenotrophomonas rhizophila*.

**Figure 7:**
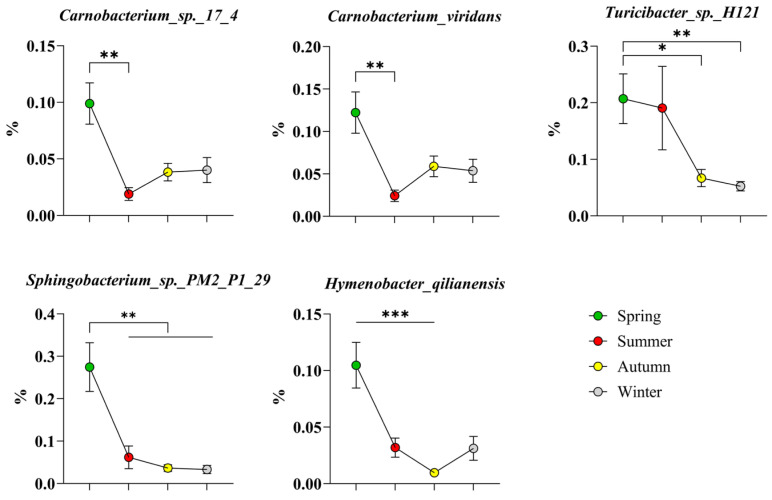
High-abundance bacteria of the phylum Bacillota in spring. The results are presented as “mean ± standard deviation”, * indicates *p* < 0.05 (significant difference), ** indicates *p* < 0.01 (highly significant difference), *** indicates *p* < 0.001.

As shown in Figure 7, the three high-abundance bacteria of the phylum *Firmicutes* in spring were *Carnobacterium* sp. 17_4, *Carnobacterium viridans*, and *Turicibacter* sp. H121, while the two species belonging to the phylum *Bacteroidota* were *Sphingobacterium* sp. PM2-P1-29 and *Hymenobacter qilianensis*.

**Figure 8:**
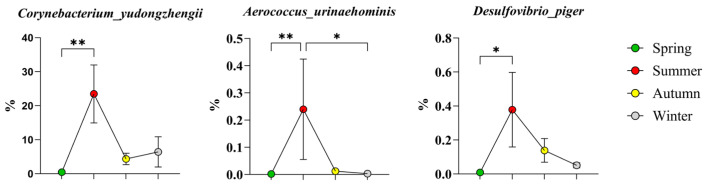
Bacteria with high abundance in summer. The results are presented as “mean ± standard deviation”, * indicates *p* < 0.05 (significant difference), ** indicates *p* < 0.01.

The high-abundance bacteria in summer were as follows (Figure 8): *Corynebacterium yudongzhengii* from the phylum *Actinobacteriota*; *Aerococcus urinaehominis* from the phylum *Firmicutes*; and *Desulfovibrio piger* from the phylum *Proteobacteria*.

**Figure 9:**
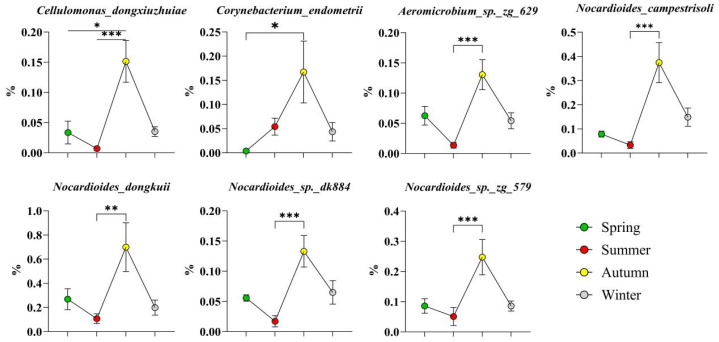
High-abundance bacteria of the phylum Actinobacteriota in autumn. The results are presented as “mean ± standard deviation”, * indicates *p* < 0.05 (significant difference), ** indicates *p* < 0.01 (highly significant difference), *** indicates *p* < 0.001.

**Figure 10:**
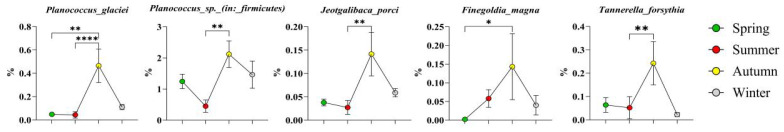
High-abundance bacteria of the phyla Firmicutes and Bacteroidetes in autumn. The results are presented as “mean ± standard deviation”, * indicates *p* < 0.05 (significant difference), ** indicates *p* < 0.01 (highly significant difference), **** indicates *p* < 0.0001.

**Figure 11:**
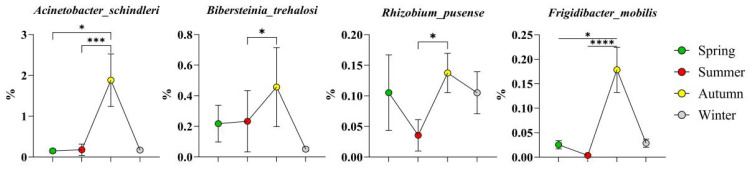
High-abundance bacteria of the phylum Proteobacteria in autumn. The results are presented as “mean ± standard deviation”, * indicates *p* < 0.05 (significant difference), *** indicates *p* < 0.001 (highly significant difference), **** indicates *p* < 0.0001.

A total of 16 high-abundance bacteria across four phyla were identified in autumn. Among them, seven species belonged to the phylum *Actinobacteriota* (Figure 9), namely, *Cellulomonas dongxiuzhuiae*, *Corynebacterium endometrii*, *Aeromicrobium* sp. zg_629, *Nocardioides campestrisoli*, *Nocardioides dongkuii*, *Nocardioides* sp. dk884, and *Nocardioides* sp. zg_579; four species belonged to the phylum *Firmicutes* (Figure 10), namely, *Planococcus glaciei*, *Planococcus* sp. in *Firmicutes*, *Jeotgalibaca porci*, and *Finegoldia magna*; only one species belonged to the phylum *Bacteroidota*: *Tannerella forsythia*; and four species belonged to the phylum *Proteobacteria* (Figure 11), namely, *Acinetobacter schindleri*, *Bibersteinia trehalosi*, *Rhizobium pusense*, and *Frigidibacter mobilis*.

**Figure 12:**
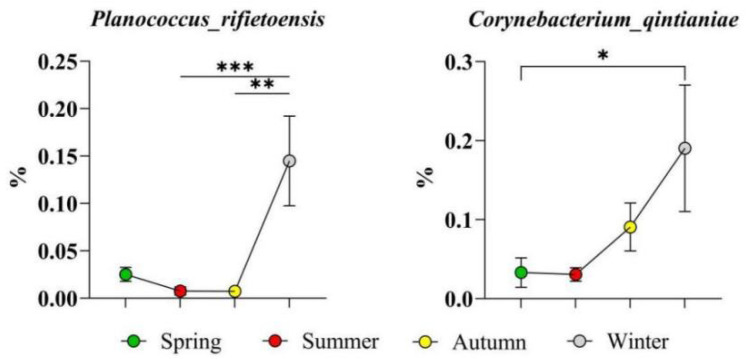
High-abundance bacteria in winter. The results are presented as “mean ± standard deviation”, * indicates *p* < 0.05 (significant difference), ** indicates *p* < 0.01 (highly significant difference), *** indicates *p* < 0.001.

High-abundance bacteria in winter included two phyla (Figure 12), namely, *Planococcus rifietoensis* from the phylum *Firmicutes* and *Corynebacterium qintianiae* from the phylum *Actinobacteriota*.

### 3.2. Metabolic Pathway Analysis

**Figure 13:**
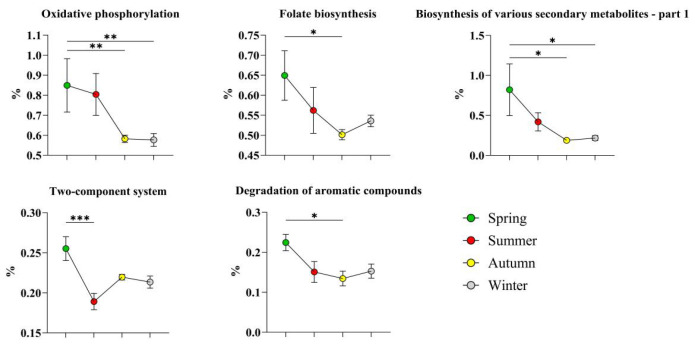
Highly abundant level 3 KEGG pathways in spring. The results are presented as “mean ± standard deviation”, * indicates *p* < 0.05 (significant difference), ** indicates *p* < 0.01 (highly significant difference), *** indicates *p* < 0.001.

The top five high-abundance level 3 KEGG pathways in spring, as shown in Figure 13, were oxidative phosphorylation, folate biosynthesis, the biosynthesis of various secondary metabolites (part 1), the two-component system, and the degradation of aromatic compounds.

**Figure 14:**
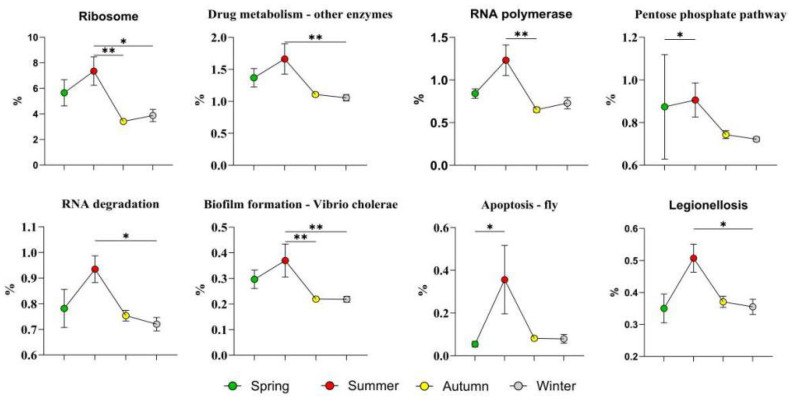
Highly abundant level 3 KEGG pathways in summer. The results are presented as “mean ± standard deviation”, * indicates *p* < 0.05 (significant difference), ** indicates *p* < 0.01.

The top eight high-abundance level 3 KEGG pathways in summer, as shown in Figure 14, were ribosome, drug metabolism-other enzymes, RNA polymerase, the pentose phosphate pathway, RNA degradation, biofilm formation-*Vibrio cholerae*, apoptosis-fly, and legionellosis.

**Figure 15:**
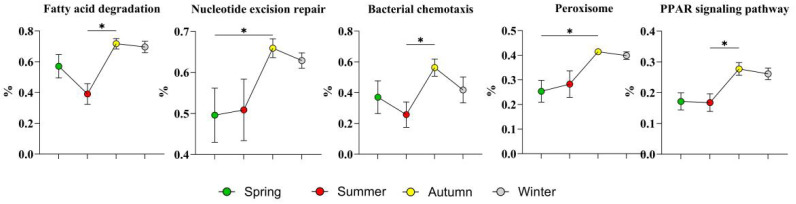
Highly abundant level 3 KEGG pathways in autumn. The results are presented as “mean ± standard deviation”, * indicates *p* < 0.05 (significant difference).

The top five high-abundance level 3 KEGG pathways in autumn, as shown in Figure 15, were fatty acid degradation, nucleotide excision repair, bacterial chemotaxis, peroxisome, and the PPAR signaling pathway.

**Figure 16:**
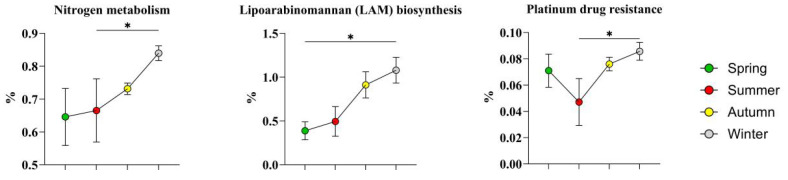
Highly abundant level 3 KEGG pathways in winter. The results are presented as “mean ± standard deviation”, * indicates *p* < 0.05 (significant difference).

The top three high-abundance level 3 KEGG pathways in winter, as shown in Figure 16, were nitrogen metabolism, lipoginomannan (LAM) biosynthesis, and platinum drug resistance.

### 3.3. Metabolite Analysis

**Figure 17:**
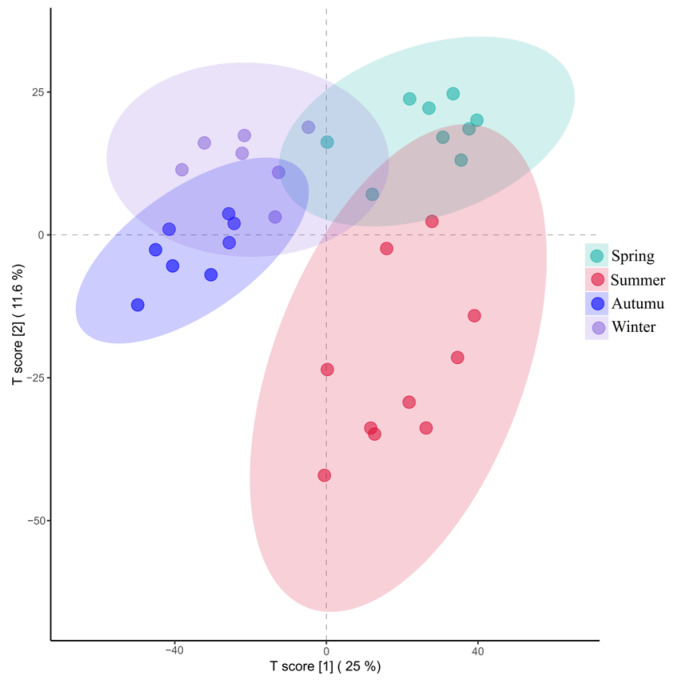
PLS-DA score plot of the skin metabolites.

PLS-DA was performed on the skin metabolites, and the results revealed that the samples from the four seasons exhibited a clear season-specific clustering pattern (Figure 17).

**Figure 18:**
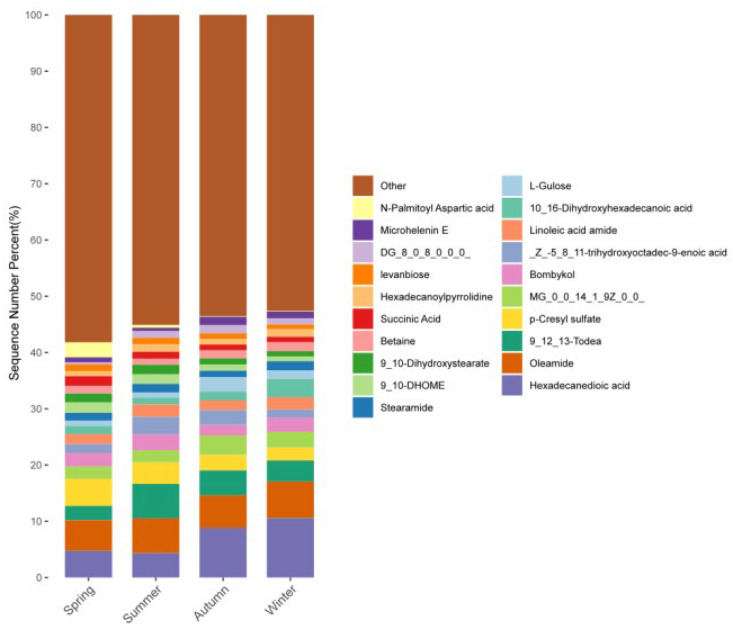
Top 20 skin metabolites by content.

A total of 3444 metabolites were detected across all four seasons. The top 20 metabolites by content, as shown in Figure 18, were hexadecanedioic acid, oleamide, 9,12,13-TriHOME/9,12,13-trihydroxy-10-octadecenoic acid, p-cresyl sulfate, 1-myristoleoyl-glycerol (MG 14:1), bombykol, (Z)-5,8,11-trihydroxyoctadec-9-enoic acid, linoleamide, 10,16-dihydroxyhexadecanoic acid, L-gulose, stearamide, 9,10-DiHOME/9,10-dihydroxy-12-octadecenoic acid, 9,10-dihydroxystearic acid, betaine, succinic acid, hexadecanoylpyrrolidine (palmitoylpyrrolidine), levanbiose, 1,2-dioctanoyl-sn-glycerol (DG 8:0/8:0), microhelenin E, and N-palmitoyl-L-aspartic acid.

**Figure 19:**
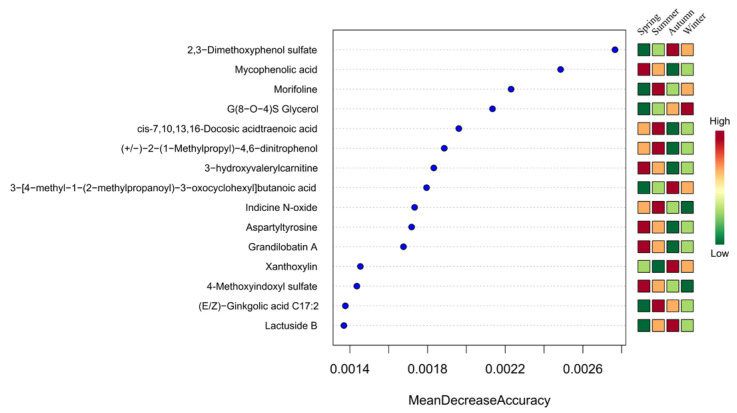
Fifteen important metabolites analyzed in the random forest model.

A random forest model was used to evaluate the importance of skin metabolites of Altay sheep across the four seasons, with the contribution of each metabolite to the seasonal classification model measured by mean decrease accuracy (MDA). This analysis was combined with the seasonal variation characteristics of metabolite relative abundance in the heatmap to screen key differentially abundant metabolites of the different seasons (Figure 19).

A total of five high-abundance metabolites were identified in spring: mycophenolic acid, 3-hydroxyvalerylcarnitine, aspartyltyrosine, grandilobatin A, and 4-methoxyindoxyl sulfate. A total of five high-abundance metabolites were identified in summer: morifoline, cis-7,10,13,16-docosatetraenoic acid, (+/−)-2-(1-methylpropyl)-4,6-dinitrophenol, indicine N-oxide, and (E/Z)-ginkgolic acid C17:2. Four high-abundance metabolites were identified in autumn: 2,3-dimethoxyphenol sulfate, 3-[4-methyl-1-(2-methylpropanoyl)-3-oxocyclohexyl]butanoic acid, xanthoxylin, and lactuside B. Only one high-abundance metabolite, G(8-O-4)S glycerol, was identified in winter.

## 4. Discussion

### 4.1. Seasonal Dynamics of Skin Microbiota Diversity

In this study, alpha diversity analysis revealed that the Chao1 and observed-features indices were significantly greater in summer than in autumn, and Faith’s phylogenetic diversity indices in spring and summer were extremely significantly greater than those in autumn and winter. These results are consistent with the pattern observed by Stothart et al. [11] in mammals, in which microbiome diversity changed significantly with season and environment. PCoA of beta diversity revealed extremely significant differences in skin bacterial community structure across the four seasons, indicating that seasonal changes, along with concomitant fluctuations in environmental factors such as temperature, humidity, vegetation type, and ultraviolet radiation intensity, directly shape the structural composition of the skin microbiota [10].

### 4.2. Taxonomic Composition and Season-Specific Bacterial Communities

In this study, *Firmicutes*, *Actinobacteriota*, *Bacteroidota*, and *Proteobacteria* were identified as the shared dominant phyla across all four seasons, which is consistent with the findings of Cholewińska et al. [26] on the skin microbiome of sheep and other ruminants. However, the composition of season-specific high-abundance bacteria markedly differed, which may be attributed to the selective pressure from seasonal environments on the skin microbial community.

Among the 39 high-abundance bacteria in spring, 35 were environmentally derived, including soil bacteria (e.g., *Arthrobacter* sp., *Nocardiopsis alba*, etc.) [27,28], aquatic bacteria (e.g., *Nocardioides aquaticus*) [29], and plant-associated bacteria (e.g., *Sphingomonas* spp.) [30,31]. This may be explained by the fact that in spring, vegetation in the lowland plain of the Irtysh River Valley begins to regreen, and sheep have frequent contact with surface soil and newly growing vegetation during feeding and resting, leading to the colonization of many soil- and plant-associated bacteria on the skin surface via direct contact. The abundance of *Cutibacterium acnes*, a core commensal bacterium in the sebaceous gland niche of mammalian skin [32], was significantly greater in spring than in other seasons. This may be due to rising temperatures in spring promoting a marked recovery of sebaceous gland function in sheep skin, providing sufficient triglyceride carbon sources for this bacterium [33]. Moreover, the relatively low environmental humidity in spring pastures results in a dry skin surface, providing a suitable colonization environment for *C. acnes* [34]. *Psychrobacter* spp. are psychrophilic to psychrotolerant bacteria that are widely distributed in cold environments [35], and multiple species of this genus are highly abundant in spring; these species adapt to the low ambient temperature in spring, further demonstrating the selective effect of environmental temperature on the skin microbiota.

The number of high-abundance bacterial species decreased sharply in summer, with only three species showing significantly high abundance. This may be due to the strong wind–sand activity, high temperature, and low humidity in the Gobi Desert pasture in summer, which caused most of the input environmental bacteria to exist only transiently at low abundance. *Corynebacterium yudongzhengii*, the core dominant species in summer, belongs to the genus *Corynebacterium*, a core dominant group of mammalian skin that preferentially colonizes sebaceous gland-rich areas [36]. High temperatures in summer promote sebaceous gland secretion, providing sufficient carbon sources for this bacterium, which may be the main reason for its high abundance. *Aerococcus urinaehominis* [37] and *Desulfovibrio piger* [38] may be derived from cross-contamination of digestive and genitourinary tract microorganisms during grazing.

A total of 16 significantly high-abundance bacteria belonging to the phyla *Actinobacteriota*, *Firmicutes*, *Bacteroidota*, and *Proteobacteria* were identified in autumn, among which nine were environmentally derived. Bacteria of the genus *Nocardioides* (phylum *Actinobacteriota*) are widely distributed in soil, water, and various high-altitude habitats and have been confirmed to have strong radiation resistance and cold tolerance [39]. *Planococcus glaciei* (phylum *Firmicutes*) is a typical low-temperature-adapted strain [40], and *Acinetobacter schindleri* (phylum *Proteobacteria*), although it is a skin-derived strain, is also radiation tolerant. The specific enrichment of these strains in autumn may be driven by high altitude, high humidity, large diurnal temperature difference, and strong ultraviolet radiation of the autumn pasture in this study.

Moreover, two opportunistic pathogens, namely, *Bibersteinia trehalosi* and *Finegoldia magna*, were significantly enriched in the autumn skin microbiota. *Bibersteinia trehalosi* is a resident bacterium in the upper respiratory tract of ruminants and a key pathogen that causes pasteurellosis pneumonia in sheep [41]. *Finegoldia magna* is an obligate anaerobic opportunistic pathogen associated with skin and soft tissue infections [42]. The sudden temperature decrease, severe diurnal temperature fluctuation, and long-distance high-altitude transhumance in autumn may cause stress in the flock and lead to decreased immune function, which may explain the high abundance of these pathogens.

Only two high-abundance bacteria were detected in winter. Among them, *Planococcus rifietoensis* [43] is a cold-tolerant and salt-tolerant environmental bacterium isolated from high Arctic permafrost and is currently known to be metabolically active at −15 °C [43]. *Corynebacterium qintianiae* was isolated from the respiratory tract of plateau ungulates, and its cell wall contains the mycolic acid skeleton characteristic of the genus *Corynebacterium* [44], which endows it with low-temperature stability. The transfer of respiratory tract-derived microorganisms to the skin surface via direct contact between sheep in winter may explain the detection of this bacterium in the skin [36]. The fact that only two cold-tolerant bacteria were highly abundant in winter further demonstrates the dominant effect of the extremely low-temperature environment on the skin microbiota structure of Altay sheep.

### 4.3. Functional Adaptations to Seasonal Environments

A total of five pathways were significantly enriched in spring. Among them, oxidative phosphorylation is the core energy metabolic pathway for bacterial ATP synthesis coupled via the cell membrane electron transport chain. Its significant enrichment in spring may be directly related to the colonization of a large number of environmentally derived aerobic bacteria on the skin surface, which is highly consistent with the pattern reported by Bardgett and van der Putten [45], in which the aerobic metabolic pathways of soil microorganisms are fully revived after soil thawing in spring. Folate, as the core cofactor of one-carbon metabolism, is directly involved in purine and pyrimidine biosynthesis and is a key material basis for rapid bacterial proliferation [46]. In this study, the number of high-abundance bacterial species in spring was the greatest among the four seasons, and the peak microbiota diversity was consistent with the enrichment of the folate biosynthesis pathway, suggesting that the skin microbiota was in an active proliferation stage in spring.

The significant enrichment of the degradation of aromatic compounds pathway in spring may be related to the composition of the high-abundance microbiota. *Sphingomonas* spp., *Stenotrophomonas* spp., and *Pseudomonas* spp., which were highly abundant in spring, are all typical bacterial groups with high-efficiency aromatic compound degradation ability [47]. This may be attributed to the fact that vegetation regreening and the decomposition of plant residues in spring pastures release a large amount of phenolic organic compounds, which come into contact with sheep skin, and the above strains can colonize by using aromatic ring cleavage enzyme systems to convert these compounds into carbon sources. Enrichment of this pathway not only reflects the dominant characteristics of environmentally derived strains but also reveals the influence of skin surface microbiota structure on functional pathways. In addition, the high abundance of the two-component system pathway may be related to the dynamic process through which strains sense microenvironmental signals via histidine kinases and regulate interspecies interactions after the increase in skin microbiota density in spring [48].

In summer, the synergistic enrichment of the ribosome and RNA polymerase pathways may be related to the regulation of transcription and other biological processes in bacteria under heat stress. The genus *Corynebacterium*, to which the summer-dominant bacterium *C. yudongzhengii* belongs, has a genome rich in genes encoding GroEL/GroES molecular chaperones and heat shock proteins [49]. The efficient expression of these stress resistance genes is highly dependent on active ribosome synthesis and transcriptional activity, with direct functional coupling between the two. The concomitant significant enrichment of the RNA degradation pathway can achieve transcriptome quality control and reduce erroneous translation through timely degradation and recycling of damaged mRNA at high temperatures [50].

The significant enrichment of the pentose phosphate pathway may also be related to the dominant microbiota in summer. NADPH produced by this pathway is the core reducing component of the bacterial antioxidant defense system, which can effectively scavenge reactive oxygen species induced by high temperature and strong ultraviolet radiation [51]. Moreover, the biofilm formation pathway was significantly activated in the summer skin microbiota. The exopolysaccharide matrix, the core structural component of the biofilm secreted by bacteria, can form a dense and stable biofilm structure, which greatly increases the tolerance of the microbiota to multiple environmental stresses, such as dehydration, high temperature, and ultraviolet radiation [52]. In the extremely dry and hot pasture environment with ultralow humidity in summer, the biofilm structure can build a locally hydrated microenvironment for embedded bacterial cells, and its hydration protection effect is the key to maintaining bacterial strain survival under continuous desiccation stress, which is also the core mechanism for a few dominant bacteria to maintain high abundance in this harsh environment. The ecological significance of biofilm formation in this specific context is further reflected in two key aspects: on the one hand, the dense exopolysaccharide matrix can physically shield bacterial cells inside the biofilm from intense ultraviolet radiation and extreme high-temperature damage and synergize with the antioxidant system mediated by the pentose phosphate pathway to jointly enhance the overall stress resistance of the skin microbiota; on the other hand, the biofilm architecture provides a stable spatial basis for intercellular communication and metabolic cooperation of the bacterial community, which enables the sharing of stress-protective molecules such as NADPH and compatible solutes among community members. This collective defense mechanism forms a group-level survival advantage that cannot be achieved by individual planktonic cells, which is consistent with the pattern reported by Decho and Gutierrez [53] in the desert soil microbiome, in which genes related to biofilm formation are significantly upregulated during the high-temperature and dry season, further confirming that biofilm formation is the core group survival strategy of the skin microbiota of grazing sheep in response to dry and hot environments.

In autumn, the nucleotide excision repair pathway was significantly enriched, which may be related to the radiation tolerance characteristics of the high-abundance microbiota. Previous studies have shown that the intensity of UV-B radiation increases by 10~12% for every 1000 m increase in altitude [54]. UV-B-induced cyclobutane pyrimidine dimers are the main source that induces bacterial genome damage, and nucleotide excision repair is the core mechanism for removing such damage [55]. The high abundance of radiation-resistant strains and this repair pathway indicate that a high-altitude, strong ultraviolet environment increases the DNA damage repair capacity of the sheep skin microecosystem, which is consistent with the significant enrichment of DNA repair-related functional genes in metagenomic studies of alpine permafrost soil [56].

Fatty acid degradation, peroxisomes, and peroxisome proliferator-activated receptor (PPAR) signaling pathways formed a synergistically regulated lipid metabolism functional module in autumn [57]. Low-temperature-adapted strains such as *Planococcus glaciei* and *Jeotgalibaca porci*, which were significantly enriched in autumn, need to maintain cell membrane fluidity at low temperatures by regulating the proportion of unsaturated fatty acids in membrane lipids, a process that depends on the synergistic effect of fatty acid modification and β-oxidation pathways [57]. Moreover, short-chain fatty acids and oxidized fatty acid derivatives produced by microbiota lipid metabolism can act as natural ligands for PPARα/γ and regulate downstream signals of skin lipid synthesis, ceramide production, and the inflammatory response by binding to nuclear receptors in host keratinocytes [58]. Previous studies have confirmed that microbe-derived short-chain fatty acids can regulate the host mucosal immune response via the PPAR signaling axis [59], which is consistent with the results of this study.

The significant enrichment of the bacterial chemotaxis pathway may be associated with the specific enrichment of the two opportunistic pathogens *B. trehalosi* and *F. magna* in autumn. Chemotaxis is a key virulence process for pathogens to sense host signals and achieve directional migration and colonization. Pathogens can sense the gradient of amino acids and organic acids released by host tissues through methyl-accepting chemotaxis proteins to locate suitable colonization sites [60]. The large diurnal temperature difference, strong radiation, and long-distance transhumance in autumn may cause skin barrier damage, and chemical signals released from wound sites can lead to the chemotaxis of pathogenic bacteria, enabling the colonization of these strains on the skin surface and causing opportunistic infections.

Only three high-abundance metabolic pathways remained in the winter skin microbiota, which was consistent with the number of high-abundance bacterial species in this season, reflecting the restrictive effect of extremely low temperature on the metabolism of the sheep skin microbiota.

The significant enrichment of the nitrogen metabolism pathway may be related to the metabolic characteristics of the high-abundance strains in winter [61]. *Corynebacterium qintianiae* can secrete proteolytic enzymes to obtain organic nitrogen sources by hydrolyzing proteins shed from the host skin stratum corneum [61]. *Planococcus rifietoensis* can maintain nitrogen metabolism homeostasis in permafrost microenvironments with extreme nitrogen deficiency. Snow cover in winter leads to a sharp decrease in the soil nitrogen mineralization rate and an extreme scarcity of available inorganic nitrogen sources on the skin surface of the sheep. The above strains efficiently utilize organic nitrogen by activating amino acid catabolism and transaminase pathways, which is consistent with the pattern in which the microbial nitrogen mineralization rate in low-temperature alpine organic soil is significantly inhibited with decreasing temperature [62].

The enrichment of the lipoarabinomannan (LAM) biosynthesis pathway may be related to the cell wall biological characteristics of *C. qintianiae*, the dominant strain in winter [63]. The cell wall of the genus *Corynebacterium* contains a mycolic acid skeleton and an arabinogalactan structure, and LAM is anchored to this cell wall skeleton via phosphatidylinositol [63]. *Corynebacterium* spp. can increase their antifreeze ability by increasing LAM biosynthesis. Moreover, LAM can decrease the secretion of proinflammatory cytokines and enhance host immunity [63]. Levy et al. [64] confirmed that skin commensal bacteria are involved in the maintenance of skin immune homeostasis. In this study, LAM synthesized by the dominant *Corynebacterium* spp. in winter may play a role in inhibiting excessive skin inflammation and maintaining skin immune homeostasis in a low-temperature environment.

### 4.4. Skin Metabolite Profiles and Host–Microbe Interactions

A total of five key differentially abundant metabolites were identified in spring. Among these compounds, mycophenolic acid can block purine synthesis [65]. During the rapid proliferation period of the skin microbiota in spring, mycophenolic acid may be involved in maintaining the dynamic balance of the skin microecology. The production of 4-methoxyindoxyl sulfate depends on the synergistic effect of commensal bacterial tryptophanase activity and the host phase II sulfation reaction [66], which may be involved in strengthening host skin barrier defense. The enrichment of 3-hydroxyvalerylcarnitine, an intermediate product of mitochondrial fatty acid β-oxidation, may be related to the enhanced energy metabolic activity of skin cells after the temperature increases in spring [67]. Aspartyltyrosine is a dipeptide metabolite, mostly derived from the hydrolysis of stratum corneum proteins or products of bacterial protease activity, which can supplement the amino acid nutrient pool on the skin surface. Grandilobatin A is a plant-derived alkaloid, which confirms that the skin metabolite profile in spring is also affected by environmental vegetation.

The functional spectrum of the five key differentially abundant metabolites identified in summer reflects the adaptation of the skin microecology to the high temperature and low humidity of the desert pasture. cis-7,10,13,16-docosatetraenoic acid is an n-6 long-chain polyunsaturated fatty acid and is a core component of cell membrane phospholipids. Skin lipid peroxidation is intensified under high temperature, and the enrichment of this metabolite reflects that the host maintains membrane structural integrity by regulating skin lipid metabolism [68]. (E/Z)-ginkgolic acid C17:2 has broad-spectrum antibacterial and anti-inflammatory activities and can significantly inhibit the growth of a variety of Gram-positive bacteria [69]. The skin is dominated by *Corynebacterium* spp. in summer, and the enrichment of ginkgolic acid may inhibit other skin bacteria via selective bacteriostasis. Indicine N-oxide is a pyrrolizidine alkaloid N-oxide derived mainly from Boraginaceae plants, and pyrrolizidine alkaloids are important chemical defense substances produced by plants that are hepatotoxic to livestock and humans; their detection may be related to the composition of summer forage. The enrichment of morifoline and (+/−)-2-(1-methylpropyl)-4,6-dinitrophenol is also related to the specific vegetation and soil chemical components of the summer pasture [70].

Among the four key differentially abundant metabolites in autumn, xanthoxylin is a phenolic compound widely distributed in Rutaceae plants that can decrease the release of proinflammatory factors by inhibiting the NF-κB signaling pathway and has significant anti-inflammatory and antioxidant activities [71]. The specific enrichment of the two opportunistic pathogens *B. trehalosi* and *F. magna* in the autumn skin microbiota may be related to potential skin infection, and the simultaneous enrichment of xanthoxylin may form a natural anti-inflammatory barrier in the host skin microenvironment, which jointly activates the skin immune regulatory network with the enriched PPAR signaling pathway during this season. 2,3-Dimethoxyphenol sulfate is a polyphenol sulfate conjugate, a detoxification product of host phase II metabolism on environmentally derived phenolic substrates, which may be related to the gut–skin axis metabolism of polyphenols ingested by sheep through feeding and contact [72]. Lactuside B is an iridoid glycoside compound with anti-inflammatory and tissue repair activities [73]. Its enrichment in autumn may be related to the demand for skin barrier damage repair during this season. The metabolites in this season are systematically correlated with the high-altitude, high-humidity, strong ultraviolet environment and the microbiota characteristics of opportunistic pathogen enrichment.

Only one key differentially abundant metabolite, G(8-O-4)S glycerol, was identified in winter, which is completely consistent with the overall trend that microbiota species diversity and high-abundance metabolic pathways in this season decreased to the lowest level of the whole year. G(8-O-4)S glycerol is a lignan compound containing lignin structural units linked by β-O-4 ether bonds and is derived mainly from the microbial degradation of lignin or the decomposition products of plant tissues [74]. Most of the winter pasture is covered by snow, and the lignin in the withered grass and shrub residues is slowly released via microbial enzymatic hydrolysis and is enriched by direct contact and adsorption on the sheep skin during resting. As the only key metabolite in winter, it is ecologically consistent with the only two cold-tolerant dominant bacteria and three metabolic pathways remaining in winter, demonstrating the restrictive effect of the extremely cold environment on the sheep skin microecology.

### 4.5. Study Limitations and Future Directions

This study exclusively focused on the skin microbiota of Altay sheep, without collecting samples from shepherds or other animals in the grazing environment. Consequently, we are unable to directly assess potential microbial sharing between the sheep flock and humans. The present study identified multiple bacterial genera, including Staphylococcus, Corynebacterium, and Acinetobacter, which are common commensals of both human and animal skin [75]. Previous studies have demonstrated that close contact between humans and livestock may facilitate microbial exchange [76]. The high abundance of environmental-source bacteria (soil- and plant-associated) in spring suggests that, within this grazing system, environmental sources may be more significant than human sources in shaping the sheep skin microbiota. Future studies incorporating parallel sampling of shepherds, other livestock, and environmental samples would facilitate understanding of the microbial ecology of transhumance grazing systems and potential zoonotic transmission pathways.

### 4.6. Skin Microecology and Sheep Common Skin Diseases

Dermatophilosis, fungal infections, and foot rot are common skin conditions that pose a threat to sheep health, and their occurrence has been associated with some extent with the state of the skin microecosystem. The seasonal dynamics of the skin microecology characterized in this study may offer a degree of reference for future research into the prevention and control of these diseases. With respect to dermatophilosis, commensal skin microbiota are considered to play a role in limiting the colonization of Dermatophilus congolensis [77]; the observed predominance of environmentally derived bacteria in spring and the elevated abundance of opportunistic pathogens in autumn may provide a microecological reference for disease surveillance during these seasons. Regarding fungal infections, the barrier function of commensal microbiota has been linked to colonization resistance against dermatophytes [78]; the seasonal differences in skin bacterial community structure and bacteriostasis-associated metabolite levels identified in this study suggest that seasonal shifts in the skin microecosystem may contribute to variation in fungal infection susceptibility, warranting further investigation. Concerning foot rot, interdigital microbial dysbiosis has been associated with the colonization of Dichelobacter nodosus [79,80], and alterations in microbial diversity have been reported to precede clinical onset [81]; the overall contraction of the skin microecosystem observed in winter may offer some reference for research into the seasonal prevention and control of foot rot. Collectively, these findings may contribute new perspectives to the study of prevention and control strategies for these three diseases from the standpoint of skin microecological dynamics.

## 5. Conclusions

This study demonstrated that marked variations were detected in the composition of season-specific high-abundance bacteria, functional pathways, and metabolites among different seasons: in spring, environmentally derived bacteria dominated, with enrichment of proliferation-related pathways and elevated levels of metabolites associated with microbiota regulation and skin barrier defense. In summer, commensal bacteria were predominant in the skin, the biofilm formation pathway was significantly activated, and metabolites related to membrane lipid homeostasis and selective bacteriostasis increased. In autumn, radiation-resistant and cold-tolerant strains were enriched, accompanied by an elevated abundance of opportunistic pathogens; repair-related pathways were active, with synchronous increases in anti-inflammatory and tissue repair-associated metabolites. In winter, the skin microecosystem exhibited an overall contracted state, and cold-tolerant strains maintained basic activities through pathways involved in nitrogen metabolism and energy synthesis.

The seasonal dynamic patterns and season-specific adaptive characteristics of the skin microbiota of Altay sheep revealed in this study can provide a theoretical basis for investigations into the skin microecological adaptation mechanisms and seasonal health management of sheep in alpine, arid, and semi-arid pastoral regions worldwide.

This study has several limitations: it only focused on a single breed of Altay sheep, relied solely on omics association analysis, and did not perform in vitro and in vivo functional verification of core microbiota and pathways. Future research may conduct comparative studies on the skin microbiome of grazing ruminants across multiple breeds and regions, validate core functional targets, and systematically elucidate the interaction mechanisms between the host and microorganisms.

## Figures and Tables

**Table 1:** Sampling time and environmental information.

Climatic and Environmental Indicators	Spring	Summer	Autumn	Winter
Longitude	87.88° E	88.3° E	88.38° E	87.88° E
Latitude	47.37° N	47.74° N	48.02° N	47.37° N
Temperature at sampling (°C)	12.00	33.00	6.00	−14.00
Maximum temperature (°C)	16.8	33.4	12.2	−16.7
Minimum temperature (°C)	8.7	19.5	4.6	−26.9
Relative humidity (%)	40	29	78.8	65.8
Altitude (m)	514.2	729.4	2263.1	514.2
Frost-free period (d)	174	170	143	174
Annual rainfall (mm)	144.4	176.4	431.3	144.4
Atmospheric pressure (hPa)	893.8	897.5	894.5	955.1
Geomorphological characteristics	Plain valley	Gobi Desert	Alpine mountainous area	Plain valley
Date	25 April 2023	10 June 2023	15 September 2023	15 December 2023

**Table 2:** Permutational multivariate analysis of variance (PERMANOVA) similarity analysis based on Bray–Curtis distance metrics.

	Group1	Group2	Pseudo-F	*p* Value	q-Value
Bray–Curtis	Spring	Summer	2.7579	0.0020	0.0024
	Spring	Autumn	2.8448	0.0010	0.0020
	Spring	Winter	2.2823	0.0010	0.0020
	Summer	Autumn	3.0885	0.0010	0.0020
	Summer	Winter	2.6503	0.0020	0.0024
	Autumn	Winter	1.6230	0.0030	0.0030

## Data Availability

The original contributions presented in this study are included in the article. Further inquiries can be directed to the corresponding author.
